# No Difference in Postoperative Recovery Outcomes Between Opioid-Free and Opioid-Sparing Anesthesia Under Multimodal Analgesic Protocol for Video-Assisted Thoracoscopic Surgery: A Propensity Score Matching Cohort Study

**DOI:** 10.3390/jcm13216581

**Published:** 2024-11-01

**Authors:** Minju Kim, Jaewon Huh, Hoon Choi, Wonjung Hwang

**Affiliations:** Department of Anesthesiology and Pain Medicine, Seoul St. Mary’s Hospital, College of Medicine, The Catholic University of Korea, Seoul 06591, Republic of Korea; jkkpsk@gmail.com (M.K.); ether@catholic.ac.kr (J.H.); hoonie83@catholic.ac.kr (H.C.)

**Keywords:** dexmedetomidine, opioid, opioid-free anesthesia, opioid-sparing anesthesia, thoracic anesthesia, enhanced recovery after surgery (ERAS)

## Abstract

**Background:** With growing concerns about opioid-related risks, efforts to reduce opioid use throughout the perioperative period have increased. This study aimed to compare postoperative recovery outcomes between opioid-free anesthesia (OFA) and opioid-sparing anesthesia (OSA) under a multimodal analgesic protocol in video-assisted thoracoscopic surgery (VATS). **Methods:** A retrospective cohort study was conducted on 196 patients undergoing VATS from August 2019 to December 2021. Patients received either dexmedetomidine-based OFA or remifentanil-based OSA. Postoperative recovery was assessed using the Quality of Recovery-15 (QoR-15) score, opioid consumption, and pain intensity. Additionally, opioid-related complications and intraoperative hemodynamic changes were evaluated. **Results:** Both groups showed similar QoR-15 scores 24 h postoperatively (124.2 ± 7.0 vs. 123.0 ± 6.9, *p* = 0.227). Opioid consumption and pain intensity were comparable, and the incidence of opioid-related adverse events did not significantly differ between the groups. Intraoperative hypotension and bradycardia were more frequent in the OFA group, but the differences were not statistically significant. **Conclusions:** The study concluded that both OFA and OSA, when used under a multimodal analgesic protocol, provided effective postoperative recovery in patients undergoing VATS with no significant differences in outcomes.

## 1. Introduction

Opioids have traditionally been the cornerstone of anesthetic adjuvant and perioperative analgesia. However, their use is associated with various adverse effects, such as nausea, vomiting, dizziness, respiratory depression, opioid-induced hyperalgesia, and the risk of dependency. These side effects not only delay postoperative recovery but also increase healthcare costs. Moreover, recent evidence suggests that opioid dependency can develop even from short-term perioperative opioid use, raising significant concerns about their widespread use in surgical settings [1,2].

In response to these risks, there has been growing interest in reducing or eliminating intraoperative opioid use through techniques like opioid-free anesthesia (OFA) [3,4]. OFA strategies entirely avoid opioids during surgery, relying instead on non-opioid alternatives such as local anesthetics, N-methyl-D-aspartate antagonists, magnesium sulfate, and α2 adrenergic agonists, often in combination with neuraxial blocks. Previous research has shown that OFA can be effectively implemented across various surgical procedures and different patient demographics [3,5,6,7]. However, completely avoiding opioids during surgery may not be feasible or safe for all patients or procedures [8,9]. In this context, opioid-sparing anesthesia (OSA) has emerged as a practical alternative. OSA strategies minimize but do not entirely eliminate opioid use, aiming to mitigate opioid-related side effects while preserving effective pain control [3,4,8]. One of the most widely used agents in both OFA and OSA is dexmedetomidine, valued for its sedative and analgesic properties. Dexmedetomidine plays a pivotal role in both OFA and OSA protocols, serving as a selective α-2 adrenergic agonist with sedative, analgesic, and sympatholytic effects. It works by inhibiting norepinephrine release and reducing sympathetic activity in the central nervous system [10,11]. Dexmedetomidine enhances endogenous analgesic pathways, leading to an opioid-sparing effect and reducing the need for perioperative opioids. Studies have demonstrated that dexmedetomidine can modulate pain pathways and attenuate perioperative stress responses, thereby improving postoperative outcomes, including lower pain scores, reduced opioid consumption, and enhanced recovery quality [12,13].

Video-assisted thoracoscopic surgery (VATS) has gained preference for lung resections because it is associated with fewer postoperative complications and faster recovery compared to traditional open thoracotomy [14,15]. Despite its minimally invasive nature, however, VATS can still result in considerable postoperative pain due to intercostal nerve and muscle injuries, rib cage retraction, and pleural damage [16,17]. Adequate pain control is essential for improving postoperative recovery and preventing complications in thoracic surgery. The introduction of enhanced recovery after surgery (ERAS) programs has placed multimodal analgesia (MMA) at the center of perioperative pain management [18]. ERAS guidelines for lung cancer surgery specifically recommend minimizing opioid use throughout the entire perioperative period. The increasing focus on minimizing opioid use in surgery leads to the question of how intraoperative opioid reduction or elimination, within an MMA framework, affects postoperative recovery quality. Assessing these effects is crucial for determining whether opioid-free or opioid-sparing strategies better align with ERAS goals for enhanced recovery.

This study aims to compare the effects of OSA and OFA on postoperative recovery in patients undergoing VATS, within the context of an MMA protocol. We hypothesize that OSA will result in postoperative outcomes comparable to those of OFA in terms of overall recovery quality, pain scores, opioid consumption, and opioid-related complications while maintaining more stable intraoperative hemodynamics.

## 2. Materials and Methods

This retrospective observational cohort study was reported in accordance with the Strengthening the Reporting of Observational Studies in Epidemiology (STROBE) guidelines. Ethical approval was obtained from the Institutional Review Board (approval number: KC22OISI0557), and the requirement for written informed consent was waived due to the retrospective design of the study.

### 2.1. Study Population

A retrospective review of electronic medical records was conducted for patients who underwent elective VATS for lung cancer at a single tertiary care university hospital between August 2019 and December 2021. During this period, the hospital’s anesthesiology department was developing and implementing a multimodal analgesic protocol as part of the ERAS program, supported by an audited quality improvement initiative. The study included patients who received dexmedetomidine-based OFA or remifentanil-based OSA regimens sequentially implemented.

Patients were included if they were over 18 years of age, had an American Society of Anesthesiologists (ASA) physical status of II or III, and underwent elective VATS for segmentectomy or lobectomy. Exclusion criteria included pre-existing chronic pain conditions or the use of analgesic medication; opioid dependency; significant dysfunction of the cardiac, hepatic, or renal systems; significant arrhythmia or bradycardia; and patients who required conversion to open thoracotomy during the procedure or underwent combined surgeries.

### 2.2. Anesthesia and Analgesic Management

Perioperative management followed the institutional protocol, which was established in line with the thoracic ERAS guidelines [14]. Anesthetic management and surgical techniques were consistent between the two groups, except for the intraoperative use of dexmedetomidine in the OFA group and remifentanil in the OSA group. The following details outline the specific anesthesia and pain management approaches used based on institutional protocol.

Patients were premedicated with 600 mg of acetaminophen (AAP), taken orally with carbohydrate-containing fluid two hours prior to surgery. Anesthesia induction was performed with 1.5 mg/kg of propofol and 0.8 mg/kg of rocuronium. Once unconsciousness and muscle relaxation were confirmed, a left-sided double-lumen endobronchial tube (Human Broncho^®^, Insung Medical Co., Wonju, Republic of Korea) was inserted using a direct laryngoscope. Correct tube placement was verified with flexible fiberoptic bronchoscopy in both the supine and lateral decubitus positions. Mechanical ventilation during surgery followed a lung-protective strategy.

Anesthesia was maintained with 1.5 vol% (expired concentration) of sevoflurane and a continuous infusion of rocuronium at 0.5 mg/kg/h. Standard monitoring included electrocardiography, pulse oximetry, noninvasive and invasive blood pressure monitoring, end-tidal carbon dioxide, nasopharyngeal core temperature, and bispectral index (BIS; A-2000^TM^ SP, Aspect Medical Systems, Norwood, MA, USA). Anesthetic adjuvants were administered in each group according to protocol:

∗ OFA group: Patients received dexmedetomidine with an initial bolus of 0.6 μg/kg over 10 min prior to anesthesia induction, followed by a maintenance infusion of 0.5 μg/kg/h, adjusted in increments of 0.1 μg/kg/h until the intercostal block was completed.
∗ OSA group: Patients received remifentanil via a target-controlled infusion (Orchestra Base Primea^®^, Fresenius Vial, Brezins, France), with an effect-site concentration of 3–4 ng/mL during induction, followed by 1–4 ng/mL until the end of surgery.

Anesthetic adjuvants were titrated to maintain a target BIS of 40–60, ensuring that systolic blood pressure and heart rate remained within 20% of baseline values. If hemodynamic instability persisted despite dose adjustments, additional medications were administered. Atropine was provided if the heart rate dropped below 50 beats/min, and ephedrine was administered if systolic blood pressure fell below 90 mmHg. Maintenance fluid consisted of a balanced crystalloid solution, infused at a rate of 4 mL/kg/h using a warming device. Core body temperature was maintained above 36 °C throughout surgery, with forced-air warming blankets applied as needed. Upon completion of the surgery, sugammadex (2–4 mg/kg) was administered based on the train-of-four (TOF) ratio, and extubation was performed thereafter.

A consistent multimodal analgesic protocol was implemented in both groups, beginning with 600 mg of oral AAP administered before surgery. Additionally, 1 g of intravenous AAP was administered before the skin incision, followed by 30 mg of intravenous ketorolac 30 min prior to the end of surgery. Before wound closure, a thoracoscopic intercostal block was performed by the surgeon from the 4th to the 9th intercostal spaces using 20 mL of 0.25% ropivacaine. Intravenous patient-controlled analgesia (PCA) was prepared with 10–15 μg/kg fentanyl in normal saline (total volume of 100 mL), administered at a continuous infusion rate of 0 mL/h, with a 2 mL bolus and a lockout time of 10 min. Pain was assessed using a visual analog scale (VAS) every 10 min in the post-anesthetic care unit (PACU) and every 4 h in the ward. If the VAS score was ≥4 despite PCA use, rescue analgesia was administered. In the PACU, 1–2 μg/kg of intravenous fentanyl was provided, while in the ward, 30 mg of intravenous ketorolac was administered, followed by 50 mg of tramadol if additional pain relief was needed. Based on the patient’s postoperative fasting status, 1 g of intravenous AAP or 600 mg of oral AAP was administered every 6 h, and 200 mg of oral celecoxib was administered every 12 h until postoperative day 2.

In both groups, patients received 5 mg of intravenous dexamethasone after anesthesia induction and 75 μg of intravenous palonosetron before the end of surgery as prophylaxis for postoperative nausea and vomiting (PONV).

### 2.3. Data Collection and Outcome Measurements

Data were retrospectively extracted from the Research Electronic Data Capture (REDCap) database, which was specifically designed to support data collection for quality improvement and research initiatives related to the ERAS program. Data collection focused on patient demographics, intraoperative anesthetic management, postoperative pain scores, opioid consumption, and recovery outcomes.

Demographic data collected included age, sex, body measurements, ASA physical status, comorbidities, and smoking status. Clinical data included the preoperative Quality of Recovery-15 (QoR-15) score, pulmonary function, the site and the type of surgery performed, and the duration of surgery and anesthesia. These variables were used as covariates in this study.

The primary outcome was the QoR-15 score at 24 h postoperatively. The QoR-15 is a patient-centered questionnaire that assesses postoperative recovery across five dimensions: physical comfort, physical independence, psychological support, emotional state, and pain [19,20]. Scores range from 0 to 150, with higher scores indicating better recovery. The minimal clinically important difference for the QoR-15 is 8 points, and a score of 118 or higher is considered indicative of good recovery [21].

Secondary outcomes included the distribution of patients according to the quality of recovery categories at 24 h postoperatively (excellent: 136–150, good: 122–135, moderate: 90–121, poor: 0–89). Additional outcomes involved the assessment of QoR-15 scores at 48 and 72 h postoperatively. Pain intensity was evaluated using the VAS at 6, 24, and 48 h postoperatively. Total opioid consumption at these time points was converted to morphine milligram equivalents (MMEs), including both intravenous PCA and rescue analgesics. Data on the need for rescue antiemetics and rescue analgesics within 24 and 48 h postoperatively were collected. Additionally, incidences of PONV and other opioid-related adverse events, such as hypotension, dizziness, urinary retention, constipation, and respiratory depression, were recorded. Intraoperative hemodynamics were also recorded, with a focus on the number hypotension and bradycardia episodes. The frequency of atropine and ephedrine administration was also collected.

### 2.4. Statistical Analysis

Propensity scores were calculated using a logistic regression model that included the aforementioned covariates. Patients in the OFA and OSA groups were matched at a 1:1 ratio using the nearest-neighbor method without replacement, with a caliper width of 0.2 standard deviations of the logit of the propensity score. Before matching, there were 358 patients in the OFA group and 112 patients in the OSA group. After propensity score matching, 98 patients from each group were successfully matched for further analysis.

Continuous variables were compared using Student’s *t*-test or the Mann–Whitney U test, as appropriate. Categorical variables were compared using the chi-square test or Fisher’s exact test. A *p*-value of <0.05 was considered statistically significant. Statistical analyses were performed using SPSS version 25 (IBM Statistics, Chicago, IL, USA) and R 3.1.4 (the R Foundation for Statistical Computing, http://www.r-project.org, accessed on 10 March 2024).

## 3. Results

The demographic and clinical characteristics of the patients are summarized in Table 1. After propensity score matching, 98 patients from each group were matched for comparison. No significant differences in baseline characteristics were observed between the matched OFA and OSA groups. The standardized mean differences for all matched variables were below 0.1, indicating that the two groups were well-balanced in terms of the covariates. After matching, the mean dose of dexmedetomidine and remifentanil administered in each group was 0.4 ± 0.1 μg/kg/h and 0.1 ± 0.03 μg/kg/min, respectively.

Table 2 presents the QoR-15 global, domain, and item scores at 24 h postoperatively. The overall QoR-15 global score was similar between the OFA and OSA groups (125.0 ± 6.5 vs. 123.0 ± 7.2, *p* = 0.82). No significant differences were found in the individual domain scores, including pain, physical comfort, physical independence, psychological support, and emotional well-being. Similarly, no significant differences were observed in the individual item scores between the two groups.

**Figure 1:**
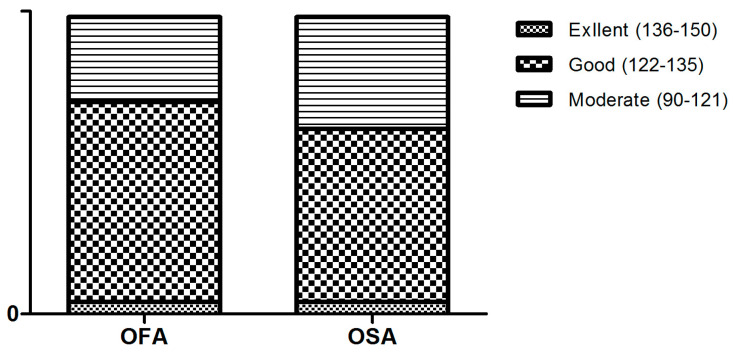
Distribution of patients according to categories of quality of recovery. OFA: opioid-free anesthesia, OSA: opioid-sparing anesthesia.

The distribution of patients across recovery categories is shown in Figure 1. The majority of patients in both groups were classified into the “good” and “moderate” categories, with no patients classified as “poor” in either group. In the OFA group, 66 patients (67.3%) were classified as having a “good” recovery, 28 patients (28.6%) as “moderate”, and 4 patients (4.1%) as “excellent”. Similarly, in the OSA group, 57 patients (58.1%) had a “good” recovery, 37 patients (37.8%) were classified as “moderate”, and 4 patients (4.1%) achieved an “excellent” recovery. The distribution of recovery categories between the groups was comparable (*p* = 0.39). Additionally, the proportion of patients achieving a good recovery (global score ≥ 118) was 80.61% in the OFA group and 75.51% in the OSA group, with no significant difference between the groups (*p* = 0.49).

The secondary outcomes related to postoperative recovery are detailed in Table 3. At 48 and 72 h postoperatively, the QoR-15 scores were similar between the OFA and OSA groups. Peak VAS scores during the 0–6, 6–24, and 24–48 h intervals after surgery were also comparable between the groups. The total amount of fentanyl included in the PCA was 1022.0 ± 105.0 μg in the OFA group and 1072.0 ± 110.0 μg in the OSA group (*p* = 0.65). The volume of PCA administered at 6, 24, and 48 h postoperatively showed no significant differences. The cumulative MME, calculated from both PCA and rescue analgesics, was not significantly different between the two groups at these time points. Additionally, the frequency of rescue analgesic administration was similar between the OFA and OSA groups during the first 24 and 48 h postoperatively.

Table 4 presents perioperative adverse events, including intraoperative and postoperative complications. Although not statistically significant, the OFA group exhibited more pronounced hemodynamic variability. The incidence of hypotension and bradycardia was higher in the OFA group compared to that in the OSA group (29% vs. 19% for hypotension, 33% vs. 19% for bradycardia, respectively). Consequently, the frequency of ephedrine and atropine administered was also higher in the OFA group, though these differences were not statistically significant. The incidence of PONV and other opioid-related complications showed no difference between the groups at both 24 and 48 h postoperatively. Opioid-related adverse events, excluding PONV, were primarily constipation and dizziness. The frequency of rescue antiemetic administration was also similar between the two groups.

## 4. Discussion

This study aimed to compare postoperative recovery outcomes in patients undergoing VATS under either OFA or OSA within an ERAS program. Our study found no significant differences in overall postoperative recovery between the OFA and OSA groups, as both demonstrated similar global QoR-15 scores at various postoperative time points. Both strategies provided effective pain management, with comparable VAS scores and cumulative opioid consumption. The incidence of PONV and other opioid-related adverse events did not significantly differ between the groups. Although intraoperative hemodynamic instability, such as hypotension and bradycardia, occurred more frequently in the OFA group, these differences were not statistically significant and did not affect recovery outcomes.

Previous studies on the application of OFA in thoracic surgery are limited, likely due to the significant pain associated with these procedures, which complicates its implementation. Additionally, variability in outcomes may stem from differences in OFA protocols and surgical settings. Even in studies focused specifically on dexmedetomidine-based OFA for VATS, the results remain inconsistent. Several studies have demonstrated that OFA and standard opioid-containing anesthesia yield similar postoperative outcomes, consistent with our findings. A prospective randomized controlled trial showed that dexmedetomidine-based OFA did not significantly differ from remifentanil-based anesthesia in terms of postoperative pain and opioid consumption in patients undergoing thoracoscopic surgery [22]. Similarly, a retrospective propensity score matching analysis of patients undergoing VATS reported no significant difference in pain scores or opioid requirements when comparing OFA with dexmedetomidine and ketamine to anesthesia with remifentanil [23]. Another retrospective study comparing OFA with dexmedetomidine to anesthesia with sufentanil also reported no significant difference in short-term analgesic consumption [24].

However, conflicting results have also been reported. A prospective randomized controlled study indicated that OFA with dexmedetomidine and lidocaine significantly reduced both postoperative pain and opioid use compared to remifentanil-based anesthesia, leading to an improved quality of recovery (QoR-40) in patients undergoing VATS [25]. Similarly, a retrospective study found that a combination of dexmedetomidine, lidocaine, and ketamine in OFA significantly decreased pain levels and opioid consumption at both 24 and 48 h after surgery compared to opioid-containing anesthesia [26].

The differences in study outcomes may be attributed to variations in opioid administration strategies in the control groups compared with those in the OFA groups. In studies reporting similar postoperative outcomes of OFA, the control groups likely incorporated non-opioid adjuvants or nerve blocks. As a result, these opioid-based protocols more closely resembled an MMA-based OSA approach, similar to our study, rather than a strict opioid-based anesthesia. By minimizing intraoperative opioid use and implementing MMA throughout the perioperative period, these protocols were able to reduce opioid consumption while still maintaining favorable recovery outcomes. On the other hand, conflicting studies used fully opioid-based protocols, where high doses of opioids were continuously infused during surgery. This practice has been linked to opioid-induced hyperalgesia (OIH), which lowers the postoperative pain threshold and increases opioid requirements [27,28]. Our study employed a much lower dose of remifentanil (0.1 ± 0.03 μg/kg/min), well below the threshold known to induce hyperalgesia [29]. This likely contributed to the reduced risk of OIH and the comparable postoperative pain outcomes observed between the two groups.

Opioid-sparing strategies involving dexmedetomidine have demonstrated effectiveness in enhancing recovery outcomes across a range of surgeries beyond VATS. In gynecological laparoscopy, these approaches have been associated with reduced pain intensity, lower analgesic requirements, and fewer opioid-related side effects, such as PONV [30,31]. Similar benefits have been observed in laparoscopic cholecystectomy and bariatric surgeries, further illustrating the versatility and advantages of opioid-sparing strategies across various surgical domains [32,33,34,35]. In oncological surgery, the potential benefits of reducing opioid use are particularly noteworthy. Recent studies suggest that opioids may suppress immune function and bind to opioid receptors expressed on tumor cells, potentially promoting tumor growth, angiogenesis, and migration [36]. Consequently, opioid use in cancer patients may contribute to cancer recurrence and metastasis. In this context, minimizing opioid exposure through OFA or OSA protocols may offer oncological advantages, although further research is required to confirm these potential benefits.

We evaluated both OFA and OSA within the framework of an ERAS protocol for VATS. Our findings show that reducing or eliminating intraoperative opioid use can be performed effectively without compromising patient outcomes. Unlike previous studies that mainly compared OFA with fully opioid-based anesthesia, which is no longer a recommended standard, we compared opioid-free and opioid-sparing strategies while maintaining consistent MMA throughout the perioperative period. Both groups demonstrated favorable recovery outcomes, with reduced opioid consumption and lower PONV incidence compared to previous studies on VATS [37,38,39]. After implementing the ERAS program at our institution, we observed a 54% reduction in opioid use and a 63% reduction in PONV incidence, while still maintaining effective analgesia. Thus, these findings emphasize the importance of minimizing opioid use not only during surgery but also throughout the entire perioperative period, supporting the integration of opioid-sparing strategies.

Our study contributes to the growing body of evidence supporting the potential utility of dexmedetomidine in OFA protocols. While OFA has been validated as a feasible approach in various surgical settings, ongoing debates remain regarding the choice of agents. Dexmedetomidine’s analgesic and sedative properties make it a valuable agent in opioid-free approaches, and its use has been linked to reduced postoperative pain and opioid requirements in various surgical procedures [40,41,42]. Despite these benefits, its hemodynamic effects, such as hypotension and bradycardia, pose significant concerns. These effects are dose-dependent, requiring careful titration to avoid adverse events, particularly in patients with cardiovascular vulnerabilities. To ensure optimal safety and efficacy during VATS procedures, we conducted a pilot study to determine the appropriate dexmedetomidine dosing. As a result, the average dexmedetomidine dose of 0.4 ± 0.1 μg/kg/h was carefully chosen to balance effective analgesia with hemodynamic stability. This dosage was determined through the pilot study, where we explored both loading and maintenance doses that were lower than the typically recommended doses. This allowed us to maintain adequate control of pain and stability throughout the surgery. This dose was also significantly lower than the >1 μg/kg/h used in other studies, where severe bradycardia and hypotension resulted in early trial termination [43]. While we observed a higher incidence of hypotension and bradycardia in the OFA group compared to the OSA group, these events were effectively managed without clinical consequences, aligning with findings from studies that employed lower dosing regimens. Our findings underscore the importance of determining surgery-specific dexmedetomidine dosing and timing to ensure the safe and effective implementation of OFA, particularly in delicate procedures like VATS.

In this study, postoperative recovery was evaluated using the Korean version of the QoR-15 questionnaire. Modern approaches to assessing surgical recovery prioritize comprehensive measures of overall recovery quality rather than isolated metrics like pain or PONV. While the QoR-40 is a well-established tool, we selected the QoR-15 due to the higher surgical complexity and older patient demographic, where compliance might be lower. Previous studies confirm that the QoR-15 is a valid, reliable, and responsive tool for measuring postoperative recovery despite its brevity [44]. Additionally, the Korean version of the QoR-15 has been shown to be an excellent alternative to the English version in similar clinical contexts, maintaining its reliability and effectiveness [45].

This study has several limitations. First, as a retrospective analysis, it is subject to inherent biases related to patient selection and data collection. Although propensity score matching was employed to minimize these biases, residual confounding factors may still exist, and the use of propensity score matching may limit the external validity of our findings. Second, this study was conducted at a single institution with a restricted patient population and adhered to a local protocol, which may limit the generalizability of our findings. Additionally, the analysis focused exclusively on VATS, which may not reflect the anesthetic requirements of more invasive thoracic surgeries such as open thoracotomy, where higher doses of dexmedetomidine or additional analgesic interventions may be needed. Multicenter randomized controlled trials are needed to validate our results across diverse clinical settings. Third, our evaluation focused solely on the acute postoperative phase, during which pain management strategies were consistent. Future studies should explore these longer-term effects to provide a more comprehensive evaluation of the impact of OFA and OSA on postoperative outcome.

## 5. Conclusions

In conclusion, both OFA and OSA approaches within an ERAS-driven MMA protocol provide comparable postoperative recovery outcomes for patients undergoing VATS. While dexmedetomidine is effective for opioid-free anesthesia, its associated hemodynamic variability warrants cautious dosing and close monitoring. We also recommend that MMA protocols, whether incorporating OFA or OSA, continue to be implemented in clinical practice to improve postoperative recovery and minimize opioid-related risks.

## Figures and Tables

**Table 1:** Patient demographics and clinical variables.

	Before Propensity Score Matching				After Propensity Score Matching			
	OFA (n = 358)	OSA (n = 112)	SMD	*p*-Value	OFA (n = 98)	OSA (n = 98)	SMD	*p*-Value
Age (yr)	65.2 ± 10.3	63.8 ± 11.2	0.13	0.21	65.1 ± 9.8	64.9 ± 10.1	0.03	0.89
Sex (male, %)	200 (56%)	60 (54%)	0.04	0.64	55 (56%)	52 (53%)	0.03	0.79
Height (cm)	168.5 ± 8.4	169.2 ± 7.8	0.09	0.35	168.7 ± 8.2	168.9 ± 8.0	0.02	0.91
Weight (kg)	70.2 ± 12.5	71.1 ± 11.9	0.07	0.28	70.3 ± 12.3	70.7 ± 11.7	0.03	0.87
BMI (kg/m^2^)	24.5 ± 3.5	25.0 ± 3.8	0.14	0.22	24.8 ± 3.4	24.9 ± 3.5	0.02	0.92
ASA (II vs. III, %)	150 (42%)	45 (40%)	0.05	0.45	40 (41%)	39 (40%)	0.02	0.93
Comorbidity (%)								
HBP	120 (34%)	40 (36%)	0.03	0.55	35 (36%)	33 (34%)	0.01	0.92
DM	60 (17%)	20 (18%)	0.05	0.31	15 (15%)	14 (14%)	0.02	0.89
COPD	40 (11%)	10 (9%)	0.06	0.44	10 (10%)	9 (9%)	0.01	0.95
Smoking status (%)								
Current	100 (28%)	30 (27%)	0.06	0.47	28 (29%)	27 (28%)	0.02	0.87
Past	120 (33%)	40 (36%)	0.10	0.62	33 (34%)	32 (33%)	0.03	0.92
Never	138 (39%)	42 (37%)	0.08	0.23	37 (37%)	39 (39%)	0.02	0.86
Preop QoR-15	141.2 ± 5.5	142.5 ± 5.4	0.14	0.14	144.5 ± 4.5	143.8 ± 4.8	0.03	0.91
Preop FEV1 (L)	2.6 ± 0.3	2.5 ± 0.4	0.09	0.33	2.5 ± 0.3	2.5 ± 0.3	0.02	0.93
Preop DLco (% predicted)	83.5 ± 4.8	81.8 ± 5.1	0.11	0.18	84.1 ± 4.6	83.9 ± 4.7	0.04	0.88
Surgical site								
Lt, %	170 (47%)	60 (54%)	0.15	0.20	46 (47%)	45 (46%)	0.03	0.95
Rt, %	188 (53%)	52 (46%)	0.13	0.34	52 (53%)	53 (54%)	0.04	0.91
Surgery type								
Segmentectomy, %	120 (34%)	40 (36%)	0.07	0.22	35 (36%)	33 (34%)	0.02	0.90
Lobectomy, %	238 (66%)	72 (64%)	0.08	0.27	63 (64%)	65 (66%)	0.03	0.91
Anesthesia time (min)	180 ± 35	175 ± 40	0.12	0.30	178 ± 33	179 ± 35	0.03	0.89
Operation time (min)	120 ± 25	115 ± 30	0.16	0.18	118 ± 26	117 ± 28	0.04	0.92

SMD: standardized mean difference, BMI: body mass index, ASA: American Society of Anesthesiologists Physical Status Classification, HBP: hypertension, DM: diabetes mellitus, COPD: chronic obstructive pulmonary disease, QoR-15: Quality of Recovery-15, FEV1: forced expiratory volume in 1 s, DLco: diffusing capacity of the lungs for carbon monoxide. Values are expressed as number (proportion) or mean ± standard deviation.

**Table 2:** QoR-15 global, domain, and item scores at 24 h after surgery.

	OFA (n = 98)	OSA (n = 98)	*p*-Value
Global score (0 to 150)	124.2 ± 7.0	123.0 ± 6.9	0.227
Domain scores			
Pain (0 to 20)	16.2 ± 1.7	16.1 ± 1.9	0.854
Physical comfort (0 to 50)	39.1 ± 3.6	38.3 ± 3.3	0.106
Physical independence (0 to 20)	16.5 ± 2.1	16.0 ± 2.2	0.130
Psychological support (0 to 20)	16.2 ± 2.0	16.0 ± 2.5	0.490
Emotions (0 to 40)	31.9 ± 2.8	32.0 ± 3.0	0.737
Item scores			
Able to breathe easily	8.0 ± 1.7	7.7 ± 1.6	0.285
Been able to enjoy food	7.5 ± 1.8	7.4 ± 1.6	0.572
Feeling rested	8.1 ± 1.4	7.9 ± 1.4	0.355
Have had a good sleep	7.9 ± 1.4	7.9 ± 1.6	0.957
Able to look after personal hygiene	8.5 ± 1.5	8.1 ± 1.7	0.065
Able to communicate with family/friends	8.0 ± 1.5	7.9 ± 1.4	0.831
Getting support from hospital doctors/nurses	8.3 ± 1.3	8.2 ± 1.6	0.895
Able to return to work/home activities	7.9 ± 1.4	7.7 ± 1.5	0.341
Feeling comfortable and in control	8.5 ± 1.3	8.5 ± 1.3	1.000
General well-being	8.0 ± 1.3	8.1 ± 1.5	0.391
Moderate pain	7.8 ± 1.5	7.8 ± 1.2	0.744
Severe pain	8.3 ± 1.4	8.4 ± 1.5	0.932
Nausea or vomiting	7.6 ± 1.6	7.4 ± 1.6	0.371
Feeling worried or anxious	7.9 ± 1.5	8.0 ± 1.4	0.669
Feeling sad or depressed	7.5 ± 1.5	7.4 ± 1.7	0.600

Values are expressed as mean ± standard deviation.

**Table 3:** Postoperative recovery, pain score, and analgesic consumption.

	OFA (n = 98)	OSA (n = 98)	*p*-Value
QoR-15 (0–150)			
At 48 h after surgery	132.0 ± 10.8	131.0 ± 10.7	0.35
At 72 h after surgery	134.5 ± 11.2	134.0 ± 11.0	0.30
Peak VAS scores			
0–6 h after surgery	4.4 ± 1.1	4.5 ± 1.2	0.45
6–24 h after surgery	4.9 ± 1.1	5.0 ± 1.2	0.41
24–48 h after surgery	4.7 ± 1.2	4.8 ± 1.3	0.43
Cumulative MMEs (mg)			
At 6 h after surgery	3.0 ± 0.7	3.2 ± 0.8	0.65
At 24 h after surgery	16.1 ± 0.8	16.3 ± 0.9	0.69
At 48 h after surgery	30.2 ± 1.0	30.4 ± 1.2	0.70
Need for rescue analgesics (Y, %)			
0–24 h after surgery	48 (49%)	51 (52%)	0.78
24–48 h after surgery	24 (25%)	26 (27%)	0.87

QoR-15: Quality of Recovery-15, VAS: visual analog scale, MME: morphine milligram equivalents. Values are expressed as number (proportion) or mean ± standard deviation.

**Table 4:** Perioperative adverse events.

	OFA (n = 98)	OSA (n = 98)	*p*-Value
Intraoperative period			
Hypotension	28 (29%)	19 (19%)	0.18
Bradycardia	32 (33%)	19 (19%)	0.05
Frequency of ephedrine administration	19 (19%)	15 (15%)	0.58
Frequency of atropine administration	26 (27%)	15 (1%)	0.08
Postoperative period			
PONV within 24 h	15 (15%)	12 (12%)	0.68
PONV within 48 h	20 (20%)	18 (18%)	0.86
Other opioid-related adverse events within 24 h	6 (6%)	4 (4%)	0.75
Other opioid-related adverse events within 48 h	7 (7%)	5 (5%)	0.77
Need for rescue antiemetics within 24 h	13 (13%)	11 (11%)	0.83
Need for rescue antiemetics within 48 h	18 (18%)	15 (15%)	0.70

PONV: postoperative nausea and vomiting. Values are expressed as number (proportion).

## Data Availability

The data generated in this study can be shared upon reasonable request to the corresponding author.

## References

[1] Wu C.L., King A.B., Geiger T.M., Grant M.C., Grocott M.P.W., Gupta R., Hah J.M., Miller T.E., Shaw A.D., Gan T.J. (2019). American Society for Enhanced Recovery and Perioperative Quality Initiative Joint Consensus Statement on Perioperative Opioid Minimization in Opioid-Naive Patients. Anesth. Analg..

[2] Soneji N., Clarke H.A., Ko D.T., Wijeysundera D.N. (2016). Risks of Developing Persistent Opioid Use After Major Surgery. JAMA Surg..

[3] Frauenknecht J., Kirkham K.R., Jacot-Guillarmod A., Albrecht E. (2019). Analgesic impact of intra-operative opioids vs. opioid-free anaesthesia: A systematic review and meta-analysis. Anaesthesia.

[4] Beloeil H. (2019). Opioid-free anaesthesia: The need for evidence-based proofs. Anaesth. Crit. Care Pain Med..

[5] Shanthanna H., Joshi G.P. (2024). Opioid-free general anesthesia: Considerations, techniques, and limitations. Curr. Opin. Anaesthesiol..

[6] Salome A., Harkouk H., Fletcher D., Martinez V. (2021). Opioid-Free Anesthesia Benefit-Risk Balance: A Systematic Review and Meta-Analysis of Randomized Controlled Trials. J. Clin. Med..

[7] Lavand’homme P., Estebe J.P. (2018). Opioid-free anesthesia: A different regard to anesthesia practice. Curr. Opin. Anaesthesiol..

[8] Shanthanna H., Ladha K.S., Kehlet H., Joshi G.P. (2021). Perioperative Opioid Administration. Anesthesiology.

[9] Chia P.A., Cannesson M., Bui C.C.M. (2020). Opioid free anesthesia: Feasible?. Curr. Opin. Anaesthesiol..

[10] Lee S. (2019). Dexmedetomidine: Present and future directions. Korean J. Anesthesiol..

[11] Weerink M.A.S., Struys M., Hannivoort L.N., Barends C.R.M., Absalom A.R., Colin P. (2017). Clinical Pharmacokinetics and Pharmacodynamics of Dexmedetomidine. Clin. Pharmacokinet..

[12] Zhao Y., He J., Yu N., Jia C., Wang S. (2020). Mechanisms of Dexmedetomidine in Neuropathic Pain. Front. Neurosci..

[13] Kaye A.D., Chernobylsky D.J., Thakur P., Siddaiah H., Kaye R.J., Eng L.K., Harbell M.W., Lajaunie J., Cornett E.M. (2020). Dexmedetomidine in Enhanced Recovery After Surgery (ERAS) Protocols for Postoperative Pain. Curr. Pain Headache Rep..

[14] Fiorelli A., Forte S., Caronia F.P., Ferrigno F., Santini M., Petersen R.H., Fang W. (2021). Is video-assisted thoracoscopic lobectomy associated with higher overall costs compared with open surgery? Results of best evidence topic analysis. Thorac. Cancer.

[15] Agostini P., Lugg S.T., Adams K., Vartsaba N., Kalkat M.S., Rajesh P.B., Steyn R.S., Naidu B., Rushton A., Bishay E. (2017). Postoperative pulmonary complications and rehabilitation requirements following lobectomy: A propensity score matched study of patients undergoing video-assisted thoracoscopic surgery versus thoracotomydagger. Interact Cardiovasc. Thorac. Surg..

[16] Feray S., Lubach J., Joshi G.P., Bonnet F., Van de Velde M., Joshi G.P., Pogatzki-Zahn E., Van de Velde M., Schug S., PROSPECT Working Group* of the European Society of Regional Anaesthesia and Pain Therapy (2022). PROSPECT guidelines for video-assisted thoracoscopic surgery: A systematic review and procedure-specific postoperative pain management recommendations. Anaesthesia.

[17] Sun L., Mu J., Gao B., Pan Y., Yu L., Liu Y., He H. (2022). Comparison of the efficacy of ultrasound-guided erector spinae plane block and thoracic paravertebral block combined with intercostal nerve block for pain management in video-assisted thoracoscopic surgery: A prospective, randomized, controlled clinical trial. BMC Anesthesiol..

[18] Batchelor T.J.P., Rasburn N.J., Abdelnour-Berchtold E., Brunelli A., Cerfolio R.J., Gonzalez M., Ljungqvist O., Petersen R.H., Popescu W.M., Slinger P.D. (2019). Guidelines for enhanced recovery after lung surgery: Recommendations of the Enhanced Recovery After Surgery (ERAS(R)) Society and the European Society of Thoracic Surgeons (ESTS). Eur. J. Cardiothorac. Surg..

[19] Kleif J., Waage J., Christensen K.B., Gogenur I. (2018). Systematic review of the QoR-15 score, a patient- reported outcome measure measuring quality of recovery after surgery and anaesthesia. Br. J. Anaesth..

[20] Jammer I., Wickboldt N., Sander M., Smith A., Schultz M.J., Pelosi P., Leva B., Rhodes A., Hoeft A., Walder B. (2015). Standards for definitions and use of outcome measures for clinical effectiveness research in perioperative medicine: European Perioperative Clinical Outcome (EPCO) definitions: A statement from the ESA-ESICM joint taskforce on perioperative outcome measures. Eur. J. Anaesthesiol..

[21] Myles P.S., Myles D.B., Galagher W., Chew C., MacDonald N., Dennis A. (2016). Minimal Clinically Important Difference for Three Quality of Recovery Scales. Anesthesiology.

[22] Jiang Y.Y., Li Z.P., Yao M., Zhou Q.H. (2022). Standard opioid-containing versus opioid-sparing anesthesia on early postoperative recovery after video-assisted thoracic surgery: A propensity-weighted analysis. Front. Surg..

[23] Mena G.E., Zorrilla-Vaca A., Vaporciyan A., Mehran R., Lasala J.D., Williams W., Patel C., Woodward T., Kruse B., Joshi G. (2022). Intraoperative Dexmedetomidine and Ketamine Infusions in an Enhanced Recovery After Thoracic Surgery Program: A Propensity Score Matched Analysis. J. Cardiothorac. Vasc. Anesth..

[24] Larue A., Jacquet-Lagreze M., Ruste M., Tronc F., Fellahi J.L. (2022). Opioid-free anaesthesia for video-assisted thoracoscopic surgery: A retrospective cohort study with propensity score analysis. Anaesth. Crit. Care Pain Med..

[25] Wang X.R., Jia X.Y., Jiang Y.Y., Li Z.P., Zhou Q.H. (2022). Opioid-free anesthesia for postoperative recovery after video-assisted thoracic surgery: A prospective, randomized controlled trial. Front. Surg..

[26] Selim J., Jarlier X., Clavier T., Boujibar F., Dusseaux M.M., Thill J., Borderelle C., Ple V., Baste J.M., Besnier E. (2022). Impact of Opioid-free Anesthesia After Video-assisted Thoracic Surgery: A Propensity Score Study. Ann. Thorac. Surg..

[27] Grape S., Kirkham K.R., Frauenknecht J., Albrecht E. (2019). Intra-operative analgesia with remifentanil vs. dexmedetomidine: A systematic review and meta-analysis with trial sequential analysis. Anaesthesia.

[28] Fletcher D., Martinez V. (2014). Opioid-induced hyperalgesia in patients after surgery: A systematic review and a meta-analysis. Br. J. Anaesth..

[29] Yu E.H., Tran D.H., Lam S.W., Irwin M.G. (2016). Remifentanil tolerance and hyperalgesia: Short-term gain, long-term pain?. Anaesthesia.

[30] Koo J.M., Chung Y.J., Lee M., Moon Y.E. (2023). Efficacy of Dexmedetomidine vs. Remifentanil for Postoperative Analgesia and Opioid-Related Side Effects after Gynecological Laparoscopy: A Prospective Randomized Controlled Trial. J. Clin. Med..

[31] Choi H., Song J.Y., Oh E.J., Chae M.S., Yu S., Moon Y.E. (2022). The Effect of Opioid-Free Anesthesia on the Quality of Recovery After Gynecological Laparoscopy: A Prospective Randomized Controlled Trial. J. Pain Res..

[32] Silva G.N., Brandao V.G., Perez M.V., Lewandrowski K.U., Fiorelli R.K.A. (2023). Effects of Dexmedetomidine on Immunomodulation and Pain Control in Videolaparoscopic Cholecystectomies: A Randomized, Two-Arm, Double-Blinded, Placebo-Controlled Trial. J. Pers. Med..

[33] Berlier J., Carabalona J.F., Tete H., Bouffard Y., Le-Goff M.C., Cerro V., Abrard S., Subtil F., Rimmele T. (2022). Effects of opioid-free anesthesia on postoperative morphine consumption after bariatric surgery. J. Clin. Anesth..

[34] Nam S.W., Oh A.Y., Koo B.W., Kim B.Y., Han J., Yoon J. (2022). Effect of Dexmedetomidine Compared to Remifentanil During Bariatric Surgery on Postoperative Nausea and Vomiting: A Retrospective Study. Obes. Surg..

[35] Bakan M., Umutoglu T., Topuz U., Uysal H., Bayram M., Kadioglu H., Salihoglu Z. (2015). Opioid-free total intravenous anesthesia with propofol, dexmedetomidine and lidocaine infusions for laparoscopic cholecystectomy: A prospective, randomized, double-blinded study. Braz. J. Anesthesiol..

[36] De Cassai A., Geraldini F., Tulgar S., Ahiskalioglu A., Mariano E.R., Dost B., Fusco P., Petroni G.M., Costa F., Navalesi P. (2022). Opioid-free anesthesia in oncologic surgery: The rules of the game. J. Anesth. Analg. Crit Care.

[37] Wu S., Gan C., Huang X., Jiang D., Xu Y., Liao Y., Ma F., Hong Y., Duan H., Lin P. (2022). Incidence and risk factors of postoperative nausea and vomiting in lung cancer patients following lobectomy and application of analgesic pumps. J. Int. Med. Res..

[38] Rajaram R., Rice D.C., Li Y., Bruera E., Liu E., Song C., Oh D.S. (2021). Postoperative opioid use after lobectomy for primary lung cancer: A propensity-matched analysis of Premier hospital data. J. Thorac. Cardiovasc. Surg..

[39] Razi S.S., Stephens-McDonnough J.A., Haq S., Fabbro M., Sanchez A.N., Epstein R.H., Villamizar N.R., Nguyen D.M. (2021). Significant reduction of postoperative pain and opioid analgesics requirement with an Enhanced Recovery After Thoracic Surgery protocol. J. Thorac. Cardiovasc. Surg..

[40] Jessen Lundorf L., Korvenius Nedergaard H., Moller A.M. (2016). Perioperative dexmedetomidine for acute pain after abdominal surgery in adults. Cochrane Database Syst. Rev..

[41] Schnabel A., Meyer-Friessem C.H., Reichl S.U., Zahn P.K., Pogatzki-Zahn E.M. (2013). Is intraoperative dexmedetomidine a new option for postoperative pain treatment? A meta-analysis of randomized controlled trials. Pain.

[42] Blaudszun G., Lysakowski C., Elia N., Tramer M.R. (2012). Effect of perioperative systemic alpha2 agonists on postoperative morphine consumption and pain intensity: Systematic review and meta-analysis of randomized controlled trials. Anesthesiology.

[43] Beloeil H., Garot M., Lebuffe G., Gerbaud A., Bila J., Cuvillon P., Dubout E., Oger S., Nadaud J., Becret A. (2021). Balanced Opioid-free Anesthesia with Dexmedetomidine versus Balanced Anesthesia with Remifentanil for Major or Intermediate Noncardiac Surgery. Anesthesiology.

[44] Myles P.S., Shulman M.A., Reilly J., Kasza J., Romero L. (2022). Measurement of quality of recovery after surgery using the 15-item quality of recovery scale: A systematic review and meta-analysis. Br. J. Anaesth..

[45] Lee J.H., Ki M., Choi S., Woo C.J., Kim D., Lim H., Kim D.C. (2021). Validity and reliability of the Korean version of the Quality of Recovery-15 questionnaire. Korean J. Anesthesiol..

